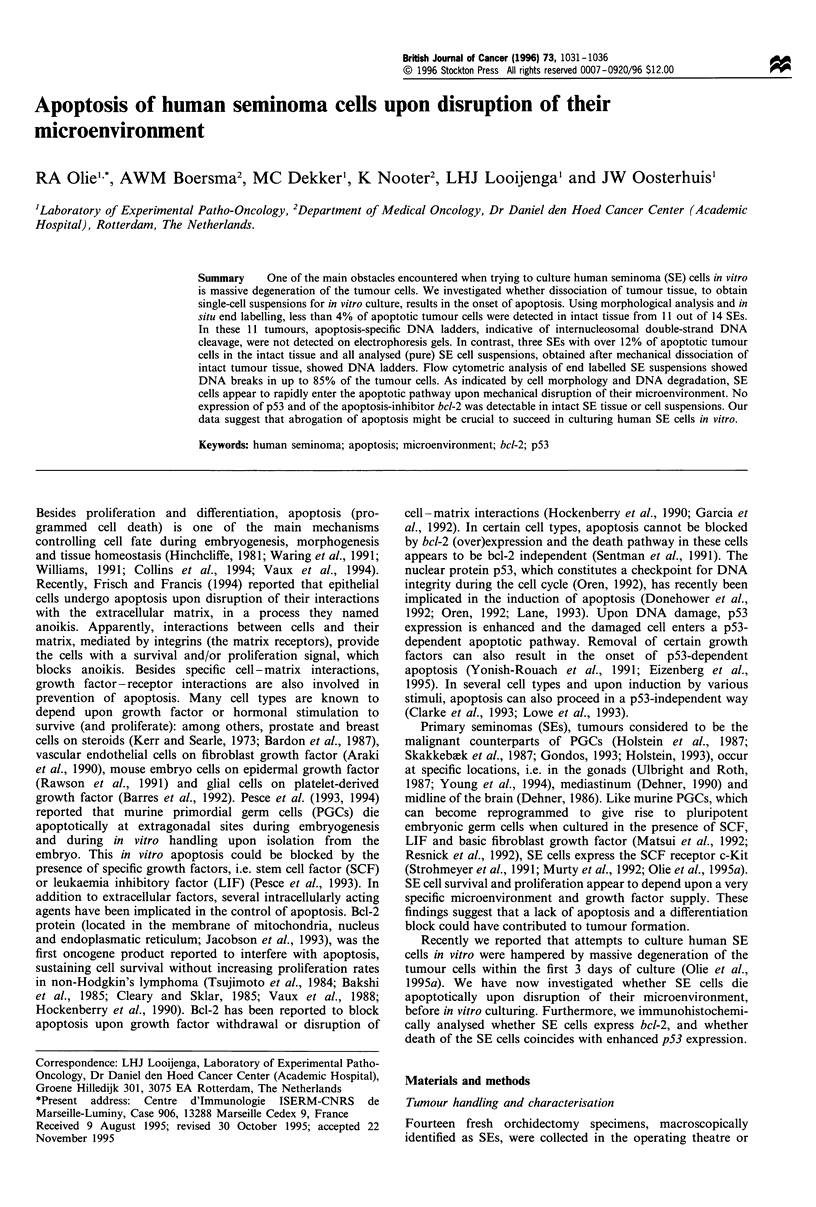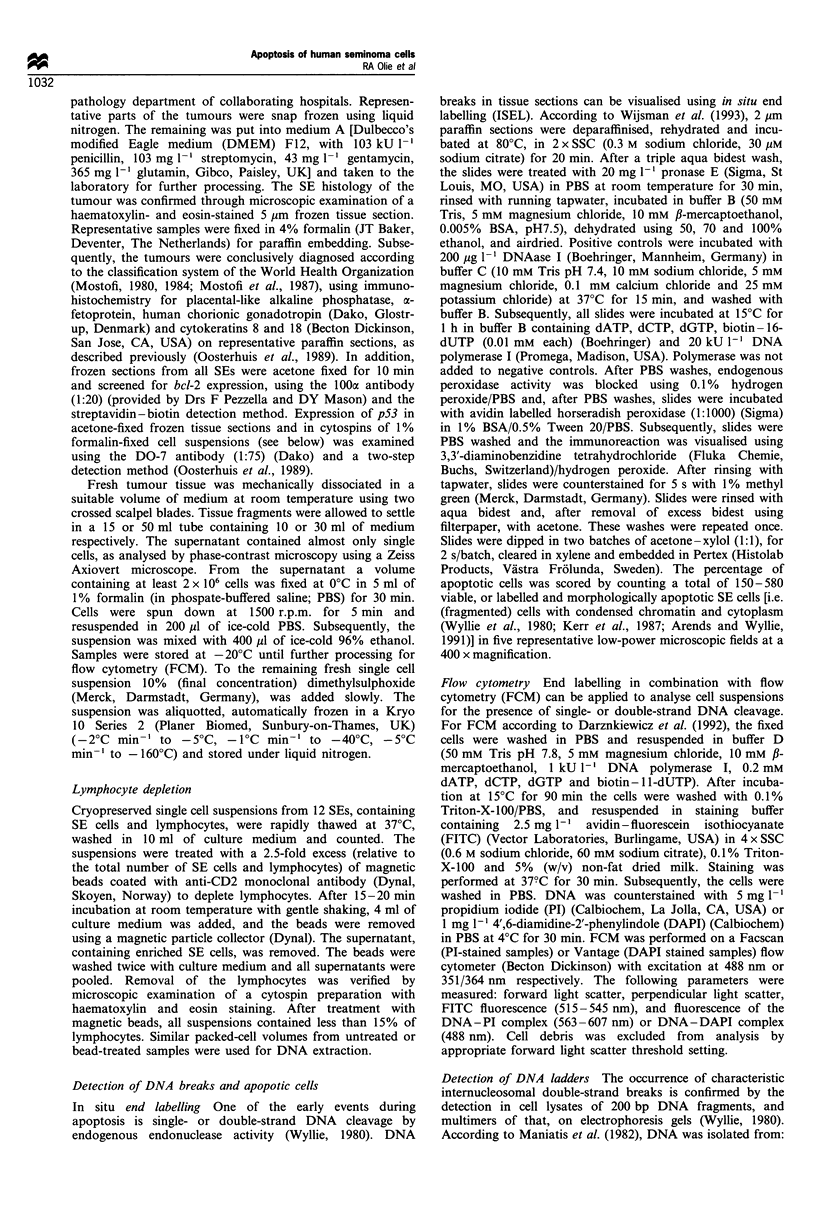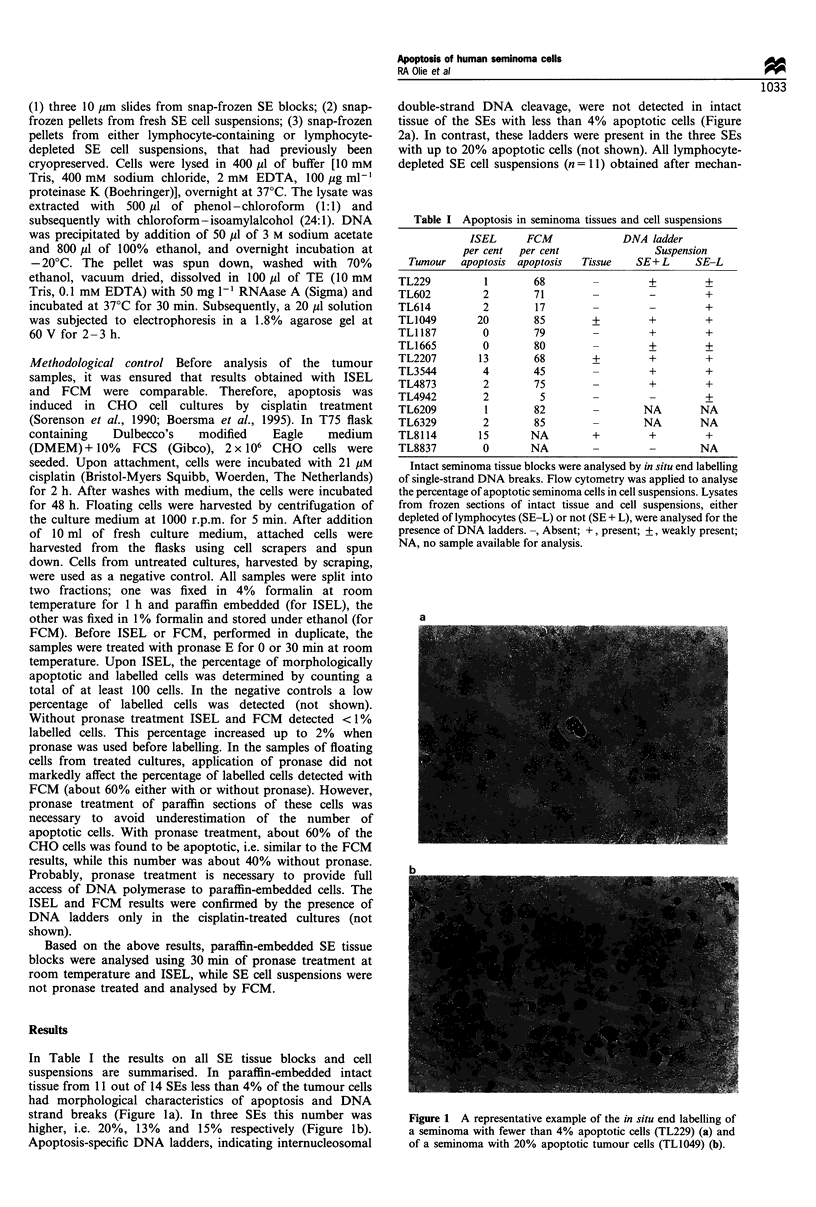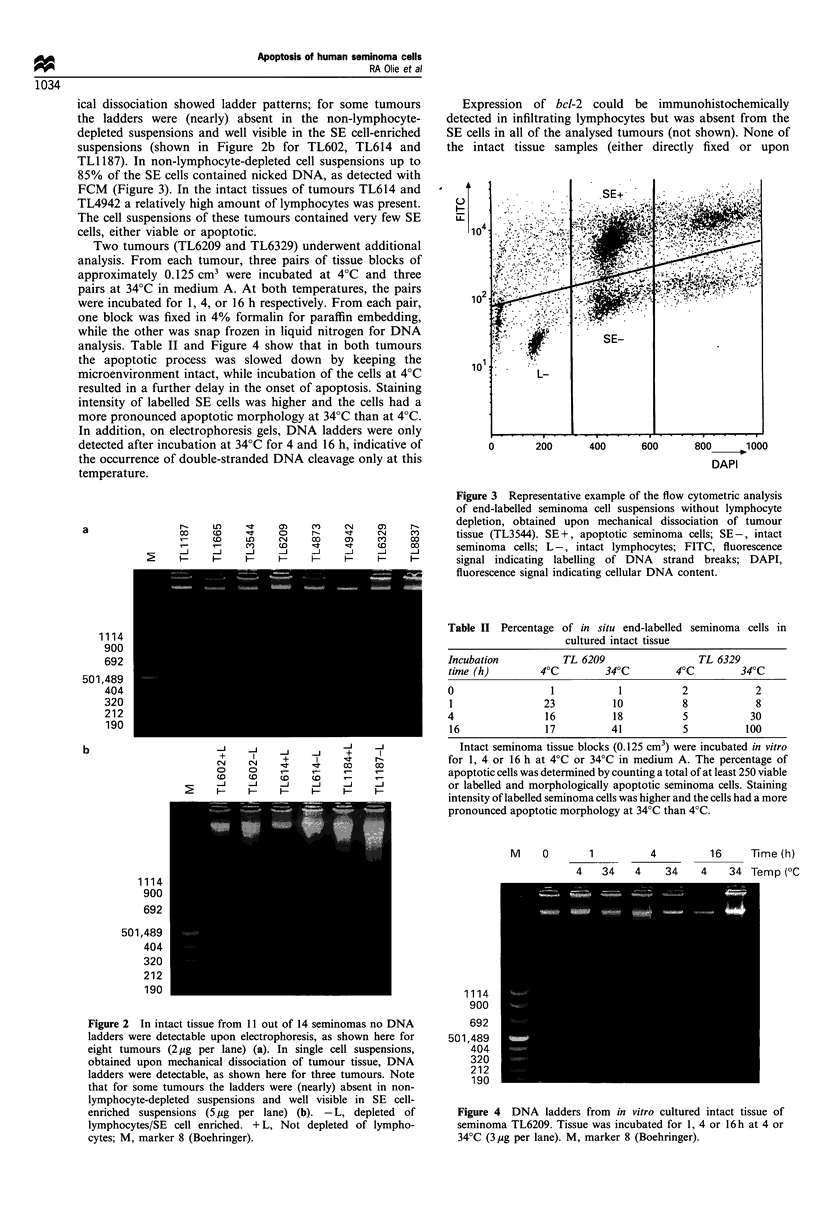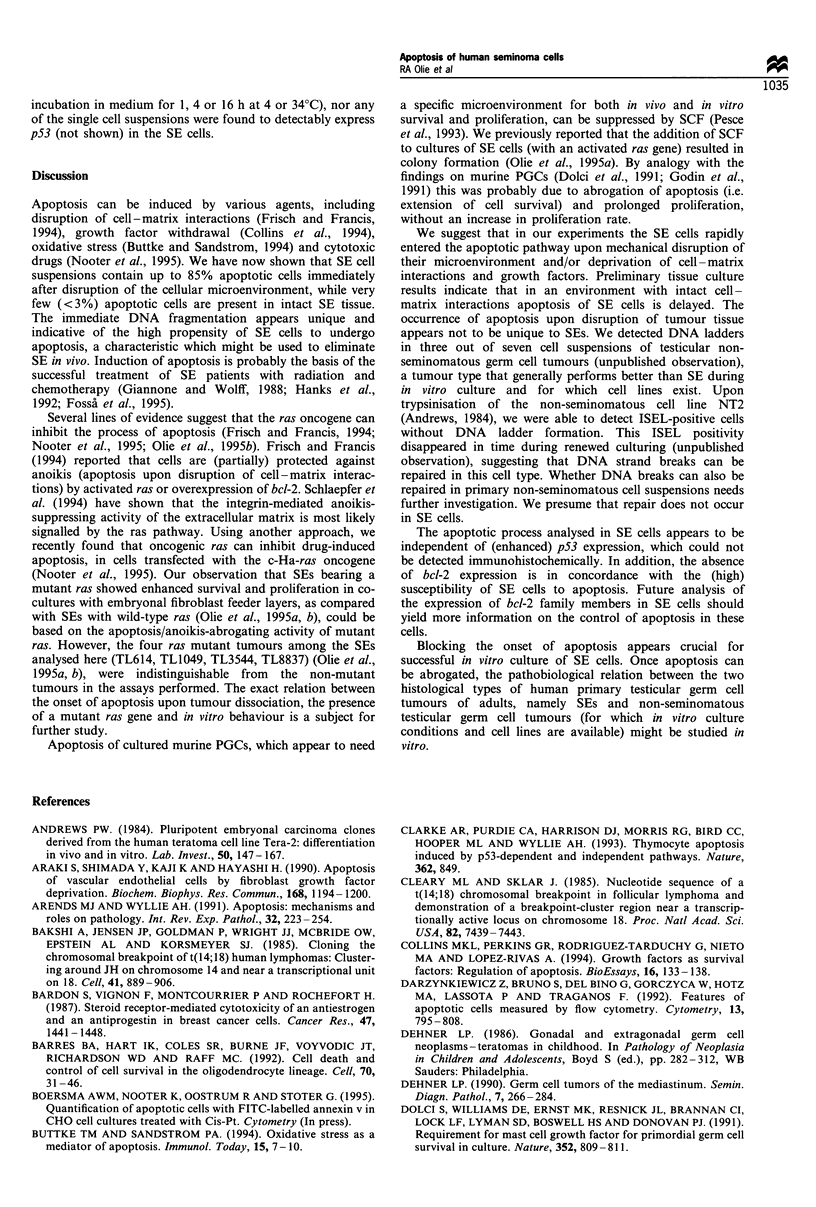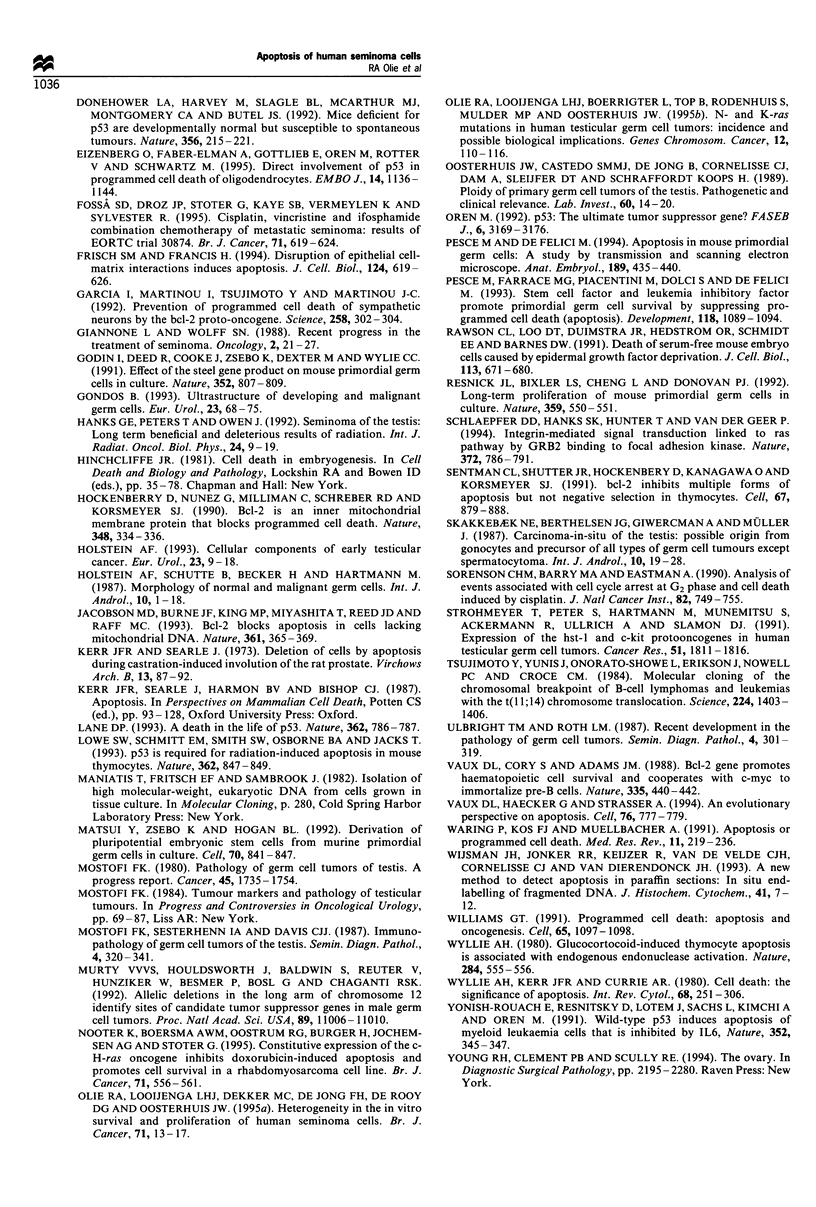# Apoptosis of human seminoma cells upon disruption of their microenvironment.

**DOI:** 10.1038/bjc.1996.200

**Published:** 1996-05

**Authors:** R. A. Olie, A. W. Boersma, M. C. Dekker, K. Nooter, L. H. Looijenga, J. W. Oosterhuis

**Affiliations:** Laboratory of Experimental Patho-Oncology, Dr Daniel den Hoed Cancer Center (Academic Hospital), Rotterdam, The Netherlands.

## Abstract

**Images:**


British Journal of Cancer (1996) 73, 1031-1036
© 1996 Stockton Press All rights reserved 0007-0920/96 $12.00

### Apoptosis of human seminoma cells upon disruption of their microenvironment

RA Olie[1,*], AWM Boersma[2], MC Dekker[1], K Nooter[2], LHJ Looijenga[1] and JW Oosterhuis[1]

[1]*Laboratory of Experimental Patho-Oncology,* [2]*Department of Medical Oncology, Dr Daniel den Hoed Cancer Center (Academic Hospital), Rotterdam, The Netherlands.*

**Summary** One of the main obstacles encountered when trying to culture human seminoma (SE) cells *in vitro* is massive degeneration of the tumour cells. We investigated whether dissociation of tumour tissue, to obtain single-cell suspensions for *in vitro* culture, results in the onset of apoptosis. Using morphological analysis and *in situ* end labelling, less than 4% of apoptotic tumour cells were detected in intact tissue from 11 out of 14 SEs. In these 11 tumours, apoptosis-specific DNA ladders, indicative of internucleosomal double-strand DNA cleavage, were not detected on electrophoresis gels. In contrast, three SEs with over 12% of apoptotic tumour cells in the intact tissue and all analysed (pure) SE cell suspensions, obtained after mechanical dissociation of intact tumour tissue, showed DNA ladders. Flow cytometric analysis of end labelled SE suspensions showed DNA breaks in up to 85% of the tumour cells. As indicated by cell morphology and DNA degradation, SE cells appear to rapidly enter the apoptotic pathway upon mechanical disruption of their microenvironment. No expression of p53 and of the apoptosis-inhibitor *bcl*-2 was detectable in intact SE tissue or cell suspensions. Our data suggest that abrogation of apoptosis might be crucial to succeed in culturing human SE cells *in vitro*.

**Keywords:** human seminoma; apoptosis; microenvironment; *bcl*-2; p53

Besides proliferation and differentiation, apoptosis (programmed cell death) is one of the main mechanisms controlling cell fate during embryogenesis, morphogenesis and tissue homeostasis (Hinchcliffe, 1981; Waring *et al.*, 1991; Williams, 1991; Collins *et al.*, 1994; Vaux *et al.*, 1994). Recently, Frisch and Francis (1994) reported that epithelial cells undergo apoptosis upon disruption of their interactions with the extracellular matrix, in a process they named anoikis. Apparently, interactions between cells and their matrix, mediated by integrins (the matrix receptors), provide the cells with a survival and/or proliferation signal, which blocks anoikis. Besides specific cell–matrix interactions, growth factor–receptor interactions are also involved in prevention of apoptosis. Many cell types are known to depend upon growth factor or hormonal stimulation to survive (and proliferate): among others, prostate and breast cells on steroids (Kerr and Searle, 1973; Bardon *et al.*, 1987), vascular endothelial cells on fibroblast growth factor (Araki *et al.*, 1990), mouse embryo cells on epidermal growth factor (Rawson *et al.*, 1991) and glial cells on platelet-derived growth factor (Barres *et al.*, 1992). Pesce *et al.* (1993, 1994) reported that murine primordial germ cells (PGCs) die apoptotically at extragonadal sites during embryogenesis and during *in vitro* handling upon isolation from the embryo. This *in vitro* apoptosis could be blocked by the presence of specific growth factors, i.e. stem cell factor (SCF) or leukaemia inhibitory factor (LIF) (Pesce *et al.*, 1993). In addition to extracellular factors, several intracellularly acting agents have been implicated in the control of apoptosis. Bcl-2 protein (located in the membrane of mitochondria, nucleus and endoplasmatic reticulum; Jacobson *et al.*, 1993), was the first oncogene product reported to interfere with apoptosis, sustaining cell survival without increasing proliferation rates in non-Hodgkin's lymphoma (Tsujimoto *et al.*, 1984; Bakshi *et al.*, 1985; Cleary and Sklar, 1985; Vaux *et al.*, 1988; Hockenberry *et al.*, 1990). Bcl-2 has been reported to block apoptosis upon growth factor withdrawal or disruption of cell–matrix interactions (Hockenberry *et al.*, 1990; Garcia *et al.*, 1992). In certain cell types, apoptosis cannot be blocked by *bcl*-2 (over)expression and the death pathway in these cells appears to be bcl-2 independent (Sentman *et al.*, 1991). The nuclear protein p53, which constitutes a checkpoint for DNA integrity during the cell cycle (Oren, 1992), has recently been implicated in the induction of apoptosis (Donehower *et al.*, 1992; Oren, 1992; Lane, 1993). Upon DNA damage, p53 expression is enhanced and the damaged cell enters a p53-dependent apoptotic pathway. Removal of certain growth factors can also result in the onset of p53-dependent apoptosis (Yonish-Rouach *et al.*, 1991; Eizenberg *et al.*, 1995). In several cell types and upon induction by various stimuli, apoptosis can also proceed in a p53-independent way (Clarke *et al.*, 1993; Lowe *et al.*, 1993).

Primary seminomas (SEs), tumours considered to be the malignant counterparts of PGCs (Holstein *et al.*, 1987; Skakkebæk *et al.*, 1987; Gondos, 1993; Holstein, 1993), occur at specific locations, i.e. in the gonads (Ulbright and Roth, 1987; Young *et al.*, 1994), mediastinum (Dehner, 1990) and midline of the brain (Dehner, 1986). Like murine PGCs, which can become reprogrammed to give rise to pluripotent embryonic germ cells when cultured in the presence of SCF, LIF and basic fibroblast growth factor (Matsui *et al.*, 1992; Resnick *et al.*, 1992), SE cells express the SCF receptor c-Kit (Strohmeyer *et al.*, 1991; Murty *et al.*, 1992; Olie *et al.*, 1995*a*). SE cell survival and proliferation appear to depend upon a very specific microenvironment and growth factor supply. These findings suggest that a lack of apoptosis and a differentiation block could have contributed to tumour formation.

Recently we reported that attempts to culture human SE cells *in vitro* were hampered by massive degeneration of the tumour cells within the first 3 days of culture (Olie *et al.*, 1995*a*). We have now investigated whether SE cells die apoptotically upon disruption of their microenvironment, before *in vitro* culturing. Furthermore, we immunohistochemically analysed whether SE cells express *bcl*-2, and whether death of the SE cells coincides with enhanced *p53* expression.

Correspondence: LHJ Looijenga, Laboratory of Experimental Patho-Oncology, Dr Daniel den Hoed Cancer Center (Academic Hospital), Groene Hilledijk 301, 3075 EA Rotterdam, The Netherlands
*Present address: Centre d'Immunologie ISERM-CNRS de Marseille-Luminy, Case 906, 13288 Marseille Cedex 9, France
Received 9 August 1995; revised 30 October 1995; accepted 22 November 1995

### Materials and methods

### *Tumour handling and characterisation*

Fourteen fresh orchidectomy specimens, macroscopically identified as SEs, were collected in the operating theatre or pathology department of collaborating hospitals. Representative parts of the tumours were snap frozen using liquid nitrogen. The remaining was put into medium A [Dulbecco's modified Eagle medium (DMEM) F12, with 103 kU $l^{-1}$ penicillin, 103 mg $l^{-1}$ streptomycin, 43 mg $l^{-1}$ gentamycin, 365 mg $l^{-1}$ glutamin, Gibco, Paisley, UK] and taken to the laboratory for further processing. The SE histology of the tumour was confirmed through microscopic examination of a haematoxylin- and eosin-stained 5 μm frozen tissue section. Representative samples were fixed in 4% formalin (JT Baker, Deventer, The Netherlands) for paraffin embedding. Subsequently, the tumours were conclusively diagnosed according to the classification system of the World Health Organization (Mostofi, 1980, 1984; Mostofi *et al.*, 1987), using immunohistochemistry for placental-like alkaline phosphatase, α-fetoprotein, human chorionic gonadotropin (Dako, Glostrup, Denmark) and cytokeratins 8 and 18 (Becton Dickinson, San Jose, CA, USA) on representative paraffin sections, as described previously (Oosterhuis *et al.*, 1989). In addition, frozen sections from all SEs were acetone fixed for 10 min and screened for *bcl-2* expression, using the 100α antibody (1:20) (provided by Drs F Pezzella and DY Mason) and the streptavidin–biotin detection method. Expression of *p53* in acetone-fixed frozen tissue sections and in cytospins of 1% formalin-fixed cell suspensions (see below) was examined using the DO-7 antibody (1:75) (Dako) and a two-step detection method (Oosterhuis *et al.*, 1989).

Fresh tumour tissue was mechanically dissociated in a suitable volume of medium at room temperature using two crossed scalpel blades. Tissue fragments were allowed to settle in a 15 or 50 ml tube containing 10 or 30 ml of medium respectively. The supernatant contained almost only single cells, as analysed by phase-contrast microscopy using a Zeiss Axiovert microscope. From the supernatant a volume containing at least $2 \times 10^6$ cells was fixed at 0°C in 5 ml of 1% formalin (in phospate-buffered saline; PBS) for 30 min. Cells were spun down at 1500 r.p.m. for 5 min and resuspended in 200 μl of ice-cold PBS. Subsequently, the suspension was mixed with 400 μl of ice-cold 96% ethanol. Samples were stored at −20°C until further processing for flow cytometry (FCM). To the remaining fresh single cell suspension 10% (final concentration) dimethylsulphoxide (Merck, Darmstadt, Germany), was added slowly. The suspension was aliquotted, automatically frozen in a Kryo 10 Series 2 (Planer Biomed, Sunbury-on-Thames, UK) (−2°C $min^{-1}$ to −5°C, −1°C $min^{-1}$ to −40°C, −5°C $min^{-1}$ to −160°C) and stored under liquid nitrogen.

*Lymphocyte depletion*

Cryopreserved single cell suspensions from 12 SEs, containing SE cells and lymphocytes, were rapidly thawed at 37°C, washed in 10 ml of culture medium and counted. The suspensions were treated with a 2.5-fold excess (relative to the total number of SE cells and lymphocytes) of magnetic beads coated with anti-CD2 monoclonal antibody (Dynal, Skoyen, Norway) to deplete lymphocytes. After 15–20 min incubation at room temperature with gentle shaking, 4 ml of culture medium was added, and the beads were removed using a magnetic particle collector (Dynal). The supernatant, containing enriched SE cells, was removed. The beads were washed twice with culture medium and all supernatants were pooled. Removal of the lymphocytes was verified by microscopic examination of a cytospin preparation with haematoxylin and eosin staining. After treatment with magnetic beads, all suspensions contained less than 15% of lymphocytes. Similar packed-cell volumes from untreated or bead-treated samples were used for DNA extraction.

*Detection of DNA breaks and apopotic cells*

In situ *end labelling* One of the early events during apoptosis is single- or double-strand DNA cleavage by endogenous endonuclease activity (Wyllie, 1980). DNA breaks in tissue sections can be visualised using *in situ* end labelling (ISEL). According to Wijsman *et al.* (1993), 2 μm paraffin sections were deparaffinised, rehydrated and incubated at 80°C, in 2 × SSC (0.3 M sodium chloride, 30 μM sodium citrate) for 20 min. After a triple aqua bidest wash, the slides were treated with 20 mg $l^{-1}$ pronase E (Sigma, St Louis, MO, USA) in PBS at room temperature for 30 min, rinsed with running tapwater, incubated in buffer B (50 mM Tris, 5 mM magnesium chloride, 10 mM β-mercaptoethanol, 0.005% BSA, pH7.5), dehydrated using 50, 70 and 100% ethanol, and airdried. Positive controls were incubated with 200 μg $l^{-1}$ DNAase I (Boehringer, Mannheim, Germany) in buffer C (10 mM Tris pH 7.4, 10 mM sodium chloride, 5 mM magnesium chloride, 0.1 mM calcium chloride and 25 mM potassium chloride) at 37°C for 15 min, and washed with buffer B. Subsequently, all slides were incubated at 15°C for 1 h in buffer B containing dATP, dCTP, dGTP, biotin–16-dUTP (0.01 mM each) (Boehringer) and 20 kU $l^{-1}$ DNA polymerase I (Promega, Madison, USA). Polymerase was not added to negative controls. After PBS washes, endogenous peroxidase activity was blocked using 0.1% hydrogen peroxide/PBS and, after PBS washes, slides were incubated with avidin labelled horseradish peroxidase (1:1000) (Sigma) in 1% BSA/0.5% Tween 20/PBS. Subsequently, slides were PBS washed and the immunoreaction was visualised using 3,3′-diaminobenzidine tetrahydrochloride (Fluka Chemie, Buchs, Switzerland)/hydrogen peroxide. After rinsing with tapwater, slides were counterstained for 5 s with 1% methyl green (Merck, Darmstadt, Germany). Slides were rinsed with aqua bidest and, after removal of excess bidest using filterpaper, with acetone. These washes were repeated once. Slides were dipped in two batches of acetone–xylol (1:1), for 2 s/batch, cleared in xylene and embedded in Pertex (Histolab Products, Västra Frölunda, Sweden). The percentage of apoptotic cells was scored by counting a total of 150–580 viable, or labelled and morphologically apoptotic SE cells [i.e. (fragmented) cells with condensed chromatin and cytoplasm (Wyllie *et al.*, 1980; Kerr *et al.*, 1987; Arends and Wyllie, 1991)] in five representative low-power microscopic fields at a 400 × magnification.

*Flow cytometry* End labelling in combination with flow cytometry (FCM) can be applied to analyse cell suspensions for the presence of single- or double-strand DNA cleavage. For FCM according to Darznkiewicz *et al.* (1992), the fixed cells were washed in PBS and resuspended in buffer D (50 mM Tris pH 7.8, 5 mM magnesium chloride, 10 mM β-mercaptoethanol, 1 kU $l^{-1}$ DNA polymerase I, 0.2 mM dATP, dCTP, dGTP and biotin–11-dUTP). After incubation at 15°C for 90 min the cells were washed with 0.1% Triton-X-100/PBS, and resuspended in staining buffer containing 2.5 mg $l^{-1}$ avidin–fluorescein isothiocyanate (FITC) (Vector Laboratories, Burlingame, USA) in 4 × SSC (0.6 M sodium chloride, 60 mM sodium citrate), 0.1% Triton-X-100 and 5% (w/v) non-fat dried milk. Staining was performed at 37°C for 30 min. Subsequently, the cells were washed in PBS. DNA was counterstained with 5 mg $l^{-1}$ propidium iodide (PI) (Calbiochem, La Jolla, CA, USA) or 1 mg $l^{-1}$ 4′,6-diamidine-2′-phenylindole (DAPI) (Calbiochem) in PBS at 4°C for 30 min. FCM was performed on a Facscan (PI-stained samples) or Vantage (DAPI stained samples) flow cytometer (Becton Dickinson) with excitation at 488 nm or 351/364 nm respectively. The following parameters were measured: forward light scatter, perpendicular light scatter, FITC fluorescence (515–545 nm), and fluorescence of the DNA–PI complex (563–607 nm) or DNA–DAPI complex (488 nm). Cell debris was excluded from analysis by appropriate forward light scatter threshold setting.

*Detection of DNA ladders* The occurrence of characteristic internucleosomal double-strand breaks is confirmed by the detection in cell lysates of 200 bp DNA fragments, and multimers of that, on electrophoresis gels (Wyllie, 1980). According to Maniatis *et al.* (1982), DNA was isolated from:

(1) three 10 $\mu$m slides from snap-frozen SE blocks; (2) snap-frozen pellets from fresh SE cell suspensions; (3) snap-frozen pellets from either lymphocyte-containing or lymphocyte-depleted SE cell suspensions, that had previously been cryopreserved. Cells were lysed in 400 $\mu$l of buffer [10 mM Tris, 400 mM sodium chloride, 2 mM EDTA, 100 $\mu$g $ml^{-1}$ proteinase K (Boehringer)], overnight at 37°C. The lysate was extracted with 500 $\mu$l of phenol–chloroform (1:1) and subsequently with chloroform–isoamylalcohol (24:1). DNA was precipitated by addition of 50 $\mu$l of 3 M sodium acetate and 800 $\mu$l of 100% ethanol, and overnight incubation at −20°C. The pellet was spun down, washed with 70% ethanol, vacuum dried, dissolved in 100 $\mu$l of TE (10 mM Tris, 0.1 mM EDTA) with 50 mg $l^{-1}$ RNAase A (Sigma) and incubated at 37°C for 30 min. Subsequently, a 20 $\mu$l solution was subjected to electrophoresis in a 1.8% agarose gel at 60 V for 2–3 h.

*Methodological control* Before analysis of the tumour samples, it was ensured that results obtained with ISEL and FCM were comparable. Therefore, apoptosis was induced in CHO cell cultures by cisplatin treatment (Sorenson *et al.*, 1990; Boersma *et al.*, 1995). In T75 flask containing Dulbecco's modified Eagle medium (DMEM)+10% FCS (Gibco), $2\times10^6$ CHO cells were seeded. Upon attachment, cells were incubated with 21 $\mu$M cisplatin (Bristol-Myers Squibb, Woerden, The Netherlands) for 2 h. After washes with medium, the cells were incubated for 48 h. Floating cells were harvested by centrifugation of the culture medium at 1000 r.p.m. for 5 min. After addition of 10 ml of fresh culture medium, attached cells were harvested from the flasks using cell scrapers and spun down. Cells from untreated cultures, harvested by scraping, were used as a negative control. All samples were split into two fractions; one was fixed in 4% formalin at room temperature for 1 h and paraffin embedded (for ISEL), the other was fixed in 1% formalin and stored under ethanol (for FCM). Before ISEL or FCM, performed in duplicate, the samples were treated with pronase E for 0 or 30 min at room temperature. Upon ISEL, the percentage of morphologically apoptotic and labelled cells was determined by counting a total of at least 100 cells. In the negative controls a low percentage of labelled cells was detected (not shown). Without pronase treatment ISEL and FCM detected <1% labelled cells. This percentage increased up to 2% when pronase was used before labelling. In the samples of floating cells from treated cultures, application of pronase did not markedly affect the percentage of labelled cells detected with FCM (about 60% either with or without pronase). However, pronase treatment of paraffin sections of these cells was necessary to avoid underestimation of the number of apoptotic cells. With pronase treatment, about 60% of the CHO cells was found to be apoptotic, i.e. similar to the FCM results, while this number was about 40% without pronase. Probably, pronase treatment is necessary to provide full access of DNA polymerase to paraffin-embedded cells. The ISEL and FCM results were confirmed by the presence of DNA ladders only in the cisplatin-treated cultures (not shown).

Based on the above results, paraffin-embedded SE tissue blocks were analysed using 30 min of pronase treatment at room temperature and ISEL, while SE cell suspensions were not pronase treated and analysed by FCM.

### Results

In Table I the results on all SE tissue blocks and cell suspensions are summarised. In paraffin-embedded intact tissue from 11 out of 14 SEs less than 4% of the tumour cells had morphological characteristics of apoptosis and DNA strand breaks (Figure 1a). In three SEs this number was higher, i.e. 20%, 13% and 15% respectively (Figure 1b). Apoptosis-specific DNA ladders, indicating internucleosomal double-strand DNA cleavage, were not detected in intact tissue of the SEs with less than 4% apoptotic cells (Figure 2a). In contrast, these ladders were present in the three SEs with up to 20% apoptotic cells (not shown). All lymphocyte-depleted SE cell suspensions ($n=11$) obtained after mechan-

**Table I** Apoptosis in seminoma tissues and cell suspensions

| | *ISEL* | *FCM* | *DNA ladder* | | |
|---|---|---|---|---|---|
| | *per cent* | *per cent* | | *Suspension* | |
| *Tumour* | *apoptosis* | *apoptosis* | *Tissue* | *SE+L* | *SE–L* |
| TL229 | 1 | 68 | – | ± | ± |
| TL602 | 2 | 71 | – | – | + |
| TL614 | 2 | 17 | – | – | + |
| TL1049 | 20 | 85 | ± | + | + |
| TL1187 | 0 | 79 | – | + | + |
| TL1665 | 0 | 80 | – | ± | ± |
| TL2207 | 13 | 68 | ± | + | + |
| TL3544 | 4 | 45 | – | + | + |
| TL4873 | 2 | 75 | – | + | + |
| TL4942 | 2 | 5 | – | – | ± |
| TL6209 | 1 | 82 | – | NA | NA |
| TL6329 | 2 | 85 | – | NA | NA |
| TL8114 | 15 | NA | + | + | + |
| TL8837 | 0 | NA | – | – | NA |

Intact seminoma tissue blocks were analysed by *in situ* end labelling of single-strand DNA breaks. Flow cytometry was applied to analyse the percentage of apoptotic seminoma cells in cell suspensions. Lysates from frozen sections of intact tissue and cell suspensions, either depleted of lymphocytes (SE–L) or not (SE+L), were analysed for the presence of DNA ladders. –, Absent; +, present; ±, weakly present; NA, no sample available for analysis.

**Figure 1** A representative example of the *in situ* end labelling of a seminoma with fewer than 4% apoptotic cells (TL229) (**a**) and of a seminoma with 20% apoptotic tumour cells (TL1049) (**b**).

ical dissociation showed ladder patterns; for some tumours the ladders were (nearly) absent in the non-lymphocyte-depleted suspensions and well visible in the SE cell-enriched suspensions (shown in Figure 2b for TL602, TL614 and TL1187). In non-lymphocyte-depleted cell suspensions up to 85% of the SE cells contained nicked DNA, as detected with FCM (Figure 3). In the intact tissues of tumours TL614 and TL4942 a relatively high amount of lymphocytes was present. The cell suspensions of these tumours contained very few SE cells, either viable or apoptotic.

Two tumours (TL6209 and TL6329) underwent additional analysis. From each tumour, three pairs of tissue blocks of approximately 0.125 $cm^3$ were incubated at 4°C and three pairs at 34°C in medium A. At both temperatures, the pairs were incubated for 1, 4, or 16 h respectively. From each pair, one block was fixed in 4% formalin for paraffin embedding, while the other was snap frozen in liquid nitrogen for DNA analysis. Table II and Figure 4 show that in both tumours the apoptotic process was slowed down by keeping the microenvironment intact, while incubation of the cells at 4°C resulted in a further delay in the onset of apoptosis. Staining intensity of labelled SE cells was higher and the cells had a more pronounced apoptotic morphology at 34°C than at 4°C. In addition, on electrophoresis gels, DNA ladders were only detected after incubation at 34°C for 4 and 16 h, indicative of the occurrence of double-stranded DNA cleavage only at this temperature.

**Figure 2** In intact tissue from 11 out of 14 seminomas no DNA ladders were detectable upon electrophoresis, as shown here for eight tumours (2 μg per lane) (**a**). In single cell suspensions, obtained upon mechanical dissociation of tumour tissue, DNA ladders were detectable, as shown here for three tumours. Note that for some tumours the ladders were (nearly) absent in non-lymphocyte-depleted suspensions and well visible in SE cell-enriched suspensions (5 μg per lane) (**b**). −L, depleted of lymphocytes/SE cell enriched. +L, Not depleted of lymphocytes; M, marker 8 (Boehringer).

Expression of *bcl*-2 could be immunohistochemically detected in infiltrating lymphocytes but was absent from the SE cells in all of the analysed tumours (not shown). None of the intact tissue samples (either directly fixed or upon

**Figure 3** Representative example of the flow cytometric analysis of end-labelled seminoma cell suspensions without lymphocyte depletion, obtained upon mechanical dissociation of tumour tissue (TL3544). SE+, apoptotic seminoma cells; SE−, intact seminoma cells; L−, intact lymphocytes; FITC, fluorescence signal indicating labelling of DNA strand breaks; DAPI, fluorescence signal indicating cellular DNA content.

**Table II** Percentage of *in situ* end-labelled seminoma cells in cultured intact tissue

| *Incubation time (h)* | *TL 6209* 4°C | *TL 6209* 34°C | *TL 6329* 4°C | *TL 6329* 34°C |
|---|---|---|---|---|
| 0 | 1 | 1 | 2 | 2 |
| 1 | 23 | 10 | 8 | 8 |
| 4 | 16 | 18 | 5 | 30 |
| 16 | 17 | 41 | 5 | 100 |

Intact seminoma tissue blocks (0.125 $cm^3$) were incubated *in vitro* for 1, 4 or 16 h at 4°C or 34°C in medium A. The percentage of apoptotic cells was determined by counting a total of at least 250 viable or labelled and morphologically apoptotic seminoma cells. Staining intensity of labelled seminoma cells was higher and the cells had a more pronounced apoptotic morphology at 34°C than 4°C.

**Figure 4** DNA ladders from *in vitro* cultured intact tissue of seminoma TL6209. Tissue was incubated for 1, 4 or 16 h at 4 or 34°C (3 μg per lane). M, marker 8 (Boehringer).

incubation in medium for 1, 4 or 16 h at 4 or 34°C), nor any of the single cell suspensions were found to detectably express *p53* (not shown) in the SE cells.

### Discussion

Apoptosis can be induced by various agents, including disruption of cell–matrix interactions (Frisch and Francis, 1994), growth factor withdrawal (Collins *et al.*, 1994), oxidative stress (Buttke and Sandstrom, 1994) and cytotoxic drugs (Nooter *et al.*, 1995). We have now shown that SE cell suspensions contain up to 85% apoptotic cells immediately after disruption of the cellular microenvironment, while very few (<3%) apoptotic cells are present in intact SE tissue. The immediate DNA fragmentation appears unique and indicative of the high propensity of SE cells to undergo apoptosis, a characteristic which might be used to eliminate SE *in vivo*. Induction of apoptosis is probably the basis of the successful treatment of SE patients with radiation and chemotherapy (Giannone and Wolff, 1988; Hanks *et al.*, 1992; Fosså *et al.*, 1995).

Several lines of evidence suggest that the *ras* oncogene can inhibit the process of apoptosis (Frisch and Francis, 1994; Nooter *et al.*, 1995; Olie *et al.*, 1995*b*). Frisch and Francis (1994) reported that cells are (partially) protected against anoikis (apoptosis upon disruption of cell–matrix interactions) by activated *ras* or overexpression of *bcl*-2. Schlaepfer *et al.* (1994) have shown that the integrin-mediated anoikis-suppressing activity of the extracellular matrix is most likely signalled by the ras pathway. Using another approach, we recently found that oncogenic *ras* can inhibit drug-induced apoptosis, in cells transfected with the c-Ha-*ras* oncogene (Nooter *et al.*, 1995). Our observation that SEs bearing a mutant *ras* showed enhanced survival and proliferation in co-cultures with embryonal fibroblast feeder layers, as compared with SEs with wild-type *ras* (Olie *et al.*, 1995*a*, *b*), could be based on the apoptosis/anoikis-abrogating activity of mutant *ras*. However, the four *ras* mutant tumours among the SEs analysed here (TL614, TL1049, TL3544, TL8837) (Olie *et al.*, 1995*a*, *b*), were indistinguishable from the non-mutant tumours in the assays performed. The exact relation between the onset of apoptosis upon tumour dissociation, the presence of a mutant *ras* gene and *in vitro* behaviour is a subject for further study.

Apoptosis of cultured murine PGCs, which appear to need a specific microenvironment for both *in vivo* and *in vitro* survival and proliferation, can be suppressed by SCF (Pesce *et al.*, 1993). We previously reported that the addition of SCF to cultures of SE cells (with an activated *ras* gene) resulted in colony formation (Olie *et al.*, 1995*a*). By analogy with the findings on murine PGCs (Dolci *et al.*, 1991; Godin *et al.*, 1991) this was probably due to abrogation of apoptosis (i.e. extension of cell survival) and prolonged proliferation, without an increase in proliferation rate.

We suggest that in our experiments the SE cells rapidly entered the apoptotic pathway upon mechanical disruption of their microenvironment and/or deprivation of cell–matrix interactions and growth factors. Preliminary tissue culture results indicate that in an environment with intact cell–matrix interactions apoptosis of SE cells is delayed. The occurrence of apoptosis upon disruption of tumour tissue appears not to be unique to SEs. We detected DNA ladders in three out of seven cell suspensions of testicular non-seminomatous germ cell tumours (unpublished observation), a tumour type that generally performs better than SE during *in vitro* culture and for which cell lines exist. Upon trypsinisation of the non-seminomatous cell line NT2 (Andrews, 1984), we were able to detect ISEL-positive cells without DNA ladder formation. This ISEL positivity disappeared in time during renewed culturing (unpublished observation), suggesting that DNA strand breaks can be repaired in this cell type. Whether DNA breaks can also be repaired in primary non-seminomatous cell suspensions needs further investigation. We presume that repair does not occur in SE cells.

The apoptotic process analysed in SE cells appears to be independent of (enhanced) *p53* expression, which could not be detected immunohistochemically. In addition, the absence of *bcl*-2 expression is in concordance with the (high) susceptibility of SE cells to apoptosis. Future analysis of the expression of *bcl*-2 family members in SE cells should yield more information on the control of apoptosis in these cells.

Blocking the onset of apoptosis appears crucial for successful *in vitro* culture of SE cells. Once apoptosis can be abrogated, the pathobiological relation between the two histological types of human primary testicular germ cell tumours of adults, namely SEs and non-seminomatous testicular germ cell tumours (for which *in vitro* culture conditions and cell lines are available) might be studied *in vitro*.

### References

ANDREWS PW. (1984). Pluripotent embryonal carcinoma clones derived from the human teratoma cell line Tera-2: differentiation in vivo and in vitro. *Lab. Invest.*, **50**, 147–167.

ARAKI S, SHIMADA Y, KAJI K AND HAYASHI H. (1990). Apoptosis of vascular endothelial cells by fibroblast growth factor deprivation. *Biochem. Biophys. Res. Commun.*, **168**, 1194–1200.

ARENDS MJ AND WYLLIE AH. (1991). Apoptosis: mechanisms and roles on pathology. *Int. Rev. Exp. Pathol.*, **32**, 223–254.

BAKSHI A, JENSEN JP, GOLDMAN P, WRIGHT JJ, MCBRIDE OW, EPSTEIN AL AND KORSMEYER SJ. (1985). Cloning the chromosomal breakpoint of t(14;18) human lymphomas: Clustering around JH on chromosome 14 and near a transcriptional unit on 18. *Cell*, **41**, 889–906.

BARDON S, VIGNON F, MONTCOURRIER P AND ROCHEFORT H. (1987). Steroid receptor-mediated cytotoxicity of an antiestrogen and an antiprogestin in breast cancer cells. *Cancer Res.*, **47**, 1441–1448.

BARRES BA, HART IK, COLES SR, BURNE JF, VOYVODIC JT, RICHARDSON WD AND RAFF MC. (1992). Cell death and control of cell survival in the oligodendrocyte lineage. *Cell*, **70**, 31–46.

BOERSMA AWM, NOOTER K, OOSTRUM R AND STOTER G. (1995). Quantification of apoptotic cells with FITC-labelled annexin v in CHO cell cultures treated with Cis-Pt. *Cytometry* (In press).

BUTTKE TM AND SANDSTROM PA. (1994). Oxidative stress as a mediator of apoptosis. *Immunol. Today*, **15**, 7–10.

CLARKE AR, PURDIE CA, HARRISON DJ, MORRIS RG, BIRD CC, HOOPER ML AND WYLLIE AH. (1993). Thymocyte apoptosis induced by p53-dependent and independent pathways. *Nature*, **362**, 849.

CLEARY ML AND SKLAR J. (1985). Nucleotide sequence of a t(14;18) chromosomal breakpoint in follicular lymphoma and demonstration of a breakpoint-cluster region near a transcriptionally active locus on chromosome 18. *Proc. Natl Acad. Sci. USA*, **82**, 7439–7443.

COLLINS MKL, PERKINS GR, RODRIGUEZ-TARDUCHY G, NIETO MA AND LOPEZ-RIVAS A. (1994). Growth factors as survival factors: Regulation of apoptosis. *BioEssays*, **16**, 133–138.

DARZYNKIEWICZ Z, BRUNO S, DEL BINO G, GORCZYCA W, HOTZ MA, LASSOTA P AND TRAGANOS F. (1992). Features of apoptotic cells measured by flow cytometry. *Cytometry*, **13**, 795–808.

DEHNER LP. (1986). Gonadal and extragonadal germ cell neoplasms–teratomas in childhood. In *Pathology of Neoplasia in Children and Adolescents*, Boyd S (ed.), pp. 282–312, WB Sauders: Philadelphia.

DEHNER LP. (1990). Germ cell tumors of the mediastinum. *Semin. Diagn. Pathol.*, **7**, 266–284.

DOLCI S, WILLIAMS DE, ERNST MK, RESNICK JL, BRANNAN CI, LOCK LF, LYMAN SD, BOSWELL HS AND DONOVAN PJ. (1991). Requirement for mast cell growth factor for primordial germ cell survival in culture. *Nature*, **352**, 809–811.

DONEHOWER LA, HARVEY M, SLAGLE BL, MCARTHUR MJ, MONTGOMERY CA AND BUTEL JS. (1992). Mice deficient for p53 are developmentally normal but susceptible to spontaneous tumours. *Nature*, **356,** 215–221.

EIZENBERG O, FABER-ELMAN A, GOTTLIEB E, OREN M, ROTTER V AND SCHWARTZ M. (1995). Direct involvement of p53 in programmed cell death of oligodendrocytes. *EMBO J.*, **14,** 1136–1144.

FOSSÅ SD, DROZ JP, STOTER G, KAYE SB, VERMEYLEN K AND SYLVESTER R. (1995). Cisplatin, vincristine and ifosphamide combination chemotherapy of metastatic seminoma: results of EORTC trial 30874. *Br. J. Cancer*, **71,** 619–624.

FRISCH SM AND FRANCIS H. (1994). Disruption of epithelial cell-matrix interactions induces apoptosis. *J. Cell. Biol.*, **124,** 619–626.

GARCIA I, MARTINOU I, TSUJIMOTO Y AND MARTINOU J-C. (1992). Prevention of programmed cell death of sympathetic neurons by the bcl-2 proto-oncogene. *Science*, **258,** 302–304.

GIANNONE L AND WOLFF SN. (1988). Recent progress in the treatment of seminoma. *Oncology*, **2,** 21–27.

GODIN I, DEED R, COOKE J, ZSEBO K, DEXTER M AND WYLIE CC. (1991). Effect of the steel gene product on mouse primordial germ cells in culture. *Nature*, **352,** 807–809.

GONDOS B. (1993). Ultrastructure of developing and malignant germ cells. *Eur. Urol.*, **23,** 68–75.

HANKS GE, PETERS T AND OWEN J. (1992). Seminoma of the testis: Long term beneficial and deleterious results of radiation. *Int. J. Radiat. Oncol. Biol. Phys.*, **24,** 9–19.

HINCHCLIFFE JR. (1981). Cell death in embryogenesis. In *Cell Death and Biology and Pathology*, Lockshin RA and Bowen ID (eds.), pp. 35–78. Chapman and Hall: New York.

HOCKENBERRY D, NUNEZ G, MILLIMAN C, SCHREBER RD AND KORSMEYER SJ. (1990). Bcl-2 is an inner mitochondrial membrane protein that blocks programmed cell death. *Nature*, **348,** 334–336.

HOLSTEIN AF. (1993). Cellular components of early testicular cancer. *Eur. Urol.*, **23,** 9–18.

HOLSTEIN AF, SCHUTTE B, BECKER H AND HARTMANN M. (1987). Morphology of normal and malignant germ cells. *Int. J. Androl.*, **10,** 1–18.

JACOBSON MD, BURNE JF, KING MP, MIYASHITA T, REED JD AND RAFF MC. (1993). Bcl-2 blocks apoptosis in cells lacking mitochondrial DNA. *Nature*, **361,** 365–369.

KERR JFR AND SEARLE J. (1973). Deletion of cells by apoptosis during castration-induced involution of the rat prostate. *Virchows Arch. B*, **13,** 87–92.

KERR JFR, SEARLE J, HARMON BV AND BISHOP CJ. (1987). Apoptosis. In *Perspectives on Mammalian Cell Death*, Potten CS (ed.), pp. 93–128, Oxford University Press: Oxford.

LANE DP. (1993). A death in the life of p53. *Nature*, **362,** 786–787.

LOWE SW, SCHMITT EM, SMITH SW, OSBORNE BA AND JACKS T. (1993). p53 is required for radiation-induced apoptosis in mouse thymocytes. *Nature*, **362,** 847–849.

MANIATIS T, FRITSCH EF AND SAMBROOK J. (1982). Isolation of high molecular-weight, eukaryotic DNA from cells grown in tissue culture. In *Molecular Cloning*, p. 280, Cold Spring Harbor Laboratory Press: New York.

MATSUI Y, ZSEBO K AND HOGAN BL. (1992). Derivation of pluripotential embryonic stem cells from murine primordial germ cells in culture. *Cell*, **70,** 841–847.

MOSTOFI FK. (1980). Pathology of germ cell tumors of testis. A progress report. *Cancer*, **45,** 1735–1754.

MOSTOFI FK. (1984). Tumour markers and pathology of testicular tumours. In *Progress and Controversies in Oncological Urology*, pp. 69–87, Liss AR: New York.

MOSTOFI FK, SESTERHENN IA AND DAVIS CJJ. (1987). Immunopathology of germ cell tumors of the testis. *Semin. Diagn. Pathol.*, **4,** 320–341.

MURTY VVVS, HOULDSWORTH J, BALDWIN S, REUTER V, HUNZIKER W, BESMER P, BOSL G AND CHAGANTI RSK. (1992). Allelic deletions in the long arm of chromosome 12 identify sites of candidate tumor suppressor genes in male germ cell tumors. *Proc. Natl Acad. Sci. USA*, **89,** 11006–11010.

NOOTER K, BOERSMA AWM, OOSTRUM RG, BURGER H, JOCHEMSEN AG AND STOTER G. (1995). Constitutive expression of the c-H-*ras* oncogene inhibits doxorubicin-induced apoptosis and promotes cell survival in a rhabdomyosarcoma cell line. *Br. J. Cancer*, **71,** 556–561.

OLIE RA, LOOIJENGA LHJ, DEKKER MC, DE JONG FH, DE ROOY DG AND OOSTERHUIS JW. (1995*a*). Heterogeneity in the in vitro survival and proliferation of human seminoma cells. *Br. J. Cancer*, **71,** 13–17.

OLIE RA, LOOIJENGA LHJ, BOERRIGTER L, TOP B, RODENHUIS S, MULDER MP AND OOSTERHUIS JW. (1995*b*). N- and K-*ras* mutations in human testicular germ cell tumors: incidence and possible biological implications. *Genes Chromosom. Cancer*, **12,** 110–116.

OOSTERHUIS JW, CASTEDO SMMJ, DE JONG B, CORNELISSE CJ, DAM A, SLEIJFER DT AND SCHRAFFORDT KOOPS H. (1989). Ploidy of primary germ cell tumors of the testis. Pathogenetic and clinical relevance. *Lab. Invest.*, **60,** 14–20.

OREN M. (1992). p53: The ultimate tumor suppressor gene? *FASEB J.*, **6,** 3169–3176.

PESCE M AND DE FELICI M. (1994). Apoptosis in mouse primordial germ cells: A study by transmission and scanning electron microscope. *Anat. Embryol.*, **189,** 435–440.

PESCE M, FARRACE MG, PIACENTINI M, DOLCI S AND DE FELICI M. (1993). Stem cell factor and leukemia inhibitory factor promote primordial germ cell survival by suppressing programmed cell death (apoptosis). *Development*, **118,** 1089–1094.

RAWSON CL, LOO DT, DUIMSTRA JR, HEDSTROM OR, SCHMIDT EE AND BARNES DW. (1991). Death of serum-free mouse embryo cells caused by epidermal growth factor deprivation. *J. Cell. Biol.*, **113,** 671–680.

RESNICK JL, BIXLER LS, CHENG L AND DONOVAN PJ. (1992). Long-term proliferation of mouse primordial germ cells in culture. *Nature*, **359,** 550–551.

SCHLAEPFER DD, HANKS SK, HUNTER T AND VAN DER GEER P. (1994). Integrin-mediated signal transduction linked to ras pathway by GRB2 binding to focal adhesion kinase. *Nature*, **372,** 786–791.

SENTMAN CL, SHUTTER JR, HOCKENBERY D, KANAGAWA O AND KORSMEYER SJ. (1991). bcl-2 inhibits multiple forms of apoptosis but not negative selection in thymocytes. *Cell*, **67,** 879–888.

SKAKKEBÆK NE, BERTHELSEN JG, GIWERCMAN A AND MÜLLER J. (1987). Carcinoma-in-situ of the testis: possible origin from gonocytes and precursor of all types of germ cell tumours except spermatocytoma. *Int. J. Androl.*, **10,** 19–28.

SORENSON CHM, BARRY MA AND EASTMAN A. (1990). Analysis of events associated with cell cycle arrest at $G_2$ phase and cell death induced by cisplatin. *J. Natl Cancer Inst.*, **82,** 749–755.

STROHMEYER T, PETER S, HARTMANN M, MUNEMITSU S, ACKERMANN R, ULLRICH A AND SLAMON DJ. (1991). Expression of the hst-1 and c-kit protooncogenes in human testicular germ cell tumors. *Cancer Res.*, **51,** 1811–1816.

TSUJIMOTO Y, YUNIS J, ONORATO-SHOWE L, ERIKSON J, NOWELL PC AND CROCE CM. (1984). Molecular cloning of the chromosomal breakpoint of B-cell lymphomas and leukemias with the t(11;14) chromosome translocation. *Science*, **224,** 1403–1406.

ULBRIGHT TM AND ROTH LM. (1987). Recent development in the pathology of germ cell tumors. *Semin. Diagn. Pathol.*, **4,** 301–319.

VAUX DL, CORY S AND ADAMS JM. (1988). Bcl-2 gene promotes haematopoietic cell survival and cooperates with c-myc to immortalize pre-B cells. *Nature*, **335,** 440–442.

VAUX DL, HAECKER G AND STRASSER A. (1994). An evolutionary perspective on apoptosis. *Cell*, **76,** 777–779.

WARING P, KOS FJ AND MUELLBACHER A. (1991). Apoptosis or programmed cell death. *Med. Res. Rev.*, **11,** 219–236.

WIJSMAN JH, JONKER RR, KEIJZER R, VAN DE VELDE CJH, CORNELISSE CJ AND VAN DIERENDONCK JH. (1993). A new method to detect apoptosis in paraffin sections: In situ end-labelling of fragmented DNA. *J. Histochem. Cytochem.*, **41,** 7–12.

WILLIAMS GT. (1991). Programmed cell death: apoptosis and oncogenesis. *Cell*, **65,** 1097–1098.

WYLLIE AH. (1980). Glucocortocoid-induced thymocyte apoptosis is associated with endogenous endonuclease activation. *Nature*, **284,** 555–556.

WYLLIE AH, KERR JFR AND CURRIE AR. (1980). Cell death: the significance of apoptosis. *Int. Rev. Cytol.*, **68,** 251–306.

YONISH-ROUACH E, RESNITSKY D, LOTEM J, SACHS L, KIMCHI A AND OREN M. (1991). Wild-type p53 induces apoptosis of myeloid leukaemia cells that is inhibited by IL6, *Nature*, **352,** 345–347.

YOUNG RH, CLEMENT PB AND SCULLY RE. (1994). The ovary. In *Diagnostic Surgical Pathology*, pp. 2195–2280. Raven Press: New York.